# Supplementary material for: A Rapid Method for Screening Pathogen-Associated Molecular Pattern-Triggered Immunity-Intensifying Microbes
Source: Plants (Basel). 2024 Aug 7;13(16):2185. doi: 10.3390/plants13162185 (PMC11359512; doi:10.3390/plants13162185)
Supplement: Supplementary file 1 [file plants-13-02185-s001.zip › plants-3104147-supplementary.pdf]

Supplementary Table S1. Primers used for plasmid construction in this study

Primer name	Sequence (5’-3’)	Target	Reference
GSL5PF	5'-GCTTATGGGACATTCTATCC -3'	At*GSL5*	This study
GSL5PR	5'-CATAGTAGCATATGGTAGATC -3'	At*GSL5*	This study
GFPPF	5'-TCTAGAATGGCTAGCAAAGG -3'	*gfp*	This study
GFPPR2	5'-CGGAGCTCTTATTTGTAGAGC-3'	*gfp*	This study
pBI-IndR	5'-CCCAGTCACGACGTTGTAAA-3'		(26)
